# Two cases of lung cancer with hemophagocytic lymphohistiocytosis caused by immune checkpoint inhibitors

**DOI:** 10.1111/1759-7714.13954

**Published:** 2021-03-30

**Authors:** Atsumasa Kurozumi, Hidenori Takahashi, Takayasu Watanabe, Yoshinobu Iwasaki

**Affiliations:** ^1^ Department of Respiratory Showa General Hospital Kodaira‐city Japan

**Keywords:** Hemophagocytic lymphohistiocytosis; Hscore, hyperferritinemia, immune checkpoint inhibitor, lung adenocarcinoma

## Abstract

We report the cases of two patients with secondary hemophagocytic lymphohistiocytosis caused by immune checkpoint inhibitors, who were diagnosed using the recently developed HScore. The first patient presented with fever, cytopenia, and elevated liver enzyme levels at 46 days post‐pembrolizumab administration. The HScore was 175. The second patient developed an immune‐related adverse event at 30 days after the final pembrolizumab dose. The HScore was 185. Hemophagocytic lymphohistiocytosis was confirmed in both patients, and corticotherapy improved their condition. It is challenging to diagnose hemophagocytic lymphohistiocytosis, particularly after development at a late stage. Our patients developed hemophagocytic lymphohistiocytosis late after immune checkpoint inhibitor administration. However, the HScore enabled us to diagnose both cases precisely and in a timely manner.

## Graphical abstract

Two cases of lung cancer developed HLH caused by immune checkpoint inhibitors. Both cases were diagnosed promptly using the HScore, which shows the effectiveness of the score for the diagnosis of secondary HLH by immune checkpoint inhibitors.

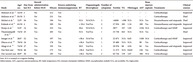

## INTRODUCTION

Recently, immune checkpoint inhibitors (ICIs) have been used to treat lung cancers. ICIs induce immune responses to cancer cells but may cause immune‐related adverse events (AEs) such as secondary hemophagocytic lymphohistiocytosis (HLH). HLH is a lethal syndrome caused by excessive inflammation, which presents as multiple organ damage. HLH is often characterized by extremely high ferritin levels, cytopenia, and elevated liver enzyme levels; however, there are many overlapping mechanisms with other systemic inflammatory syndromes, including cytokine release syndrome, sepsis, and multiple organ dysfunction syndrome. These conditions sometimes complicate the differential diagnosis of HLH. This is why there are no well‐established criteria for secondary HLH, which is often empirically diagnosed by experts. Recently, HLH has been diagnosed using the HScore, a parameter used to calculate HLH probability. Regarding HLH treatment, corticosteroids should be administered as soon as possible. The HLH‐2004 protocol suggests the effectiveness of dexamethasone and etoposide for more severe HLH patients.^1^ Treatment can be intensified by adding other immunosuppressive agents.

Here, we report two HLH cases that met the HScore cutoff value and improved with corticotherapy.

## CASE REPORT

### Case 1

A 75‐year‐old man was diagnosed with stage IV lung adenocarcinoma. He was administered carboplatin + pemetrexed as the first‐line chemotherapy and developed grade 4 AEs after two cycles; his chemotherapy was interrupted. He was admitted to our department, and pembrolizumab was initiated as the second‐line therapy.

**FIGURE 1:**
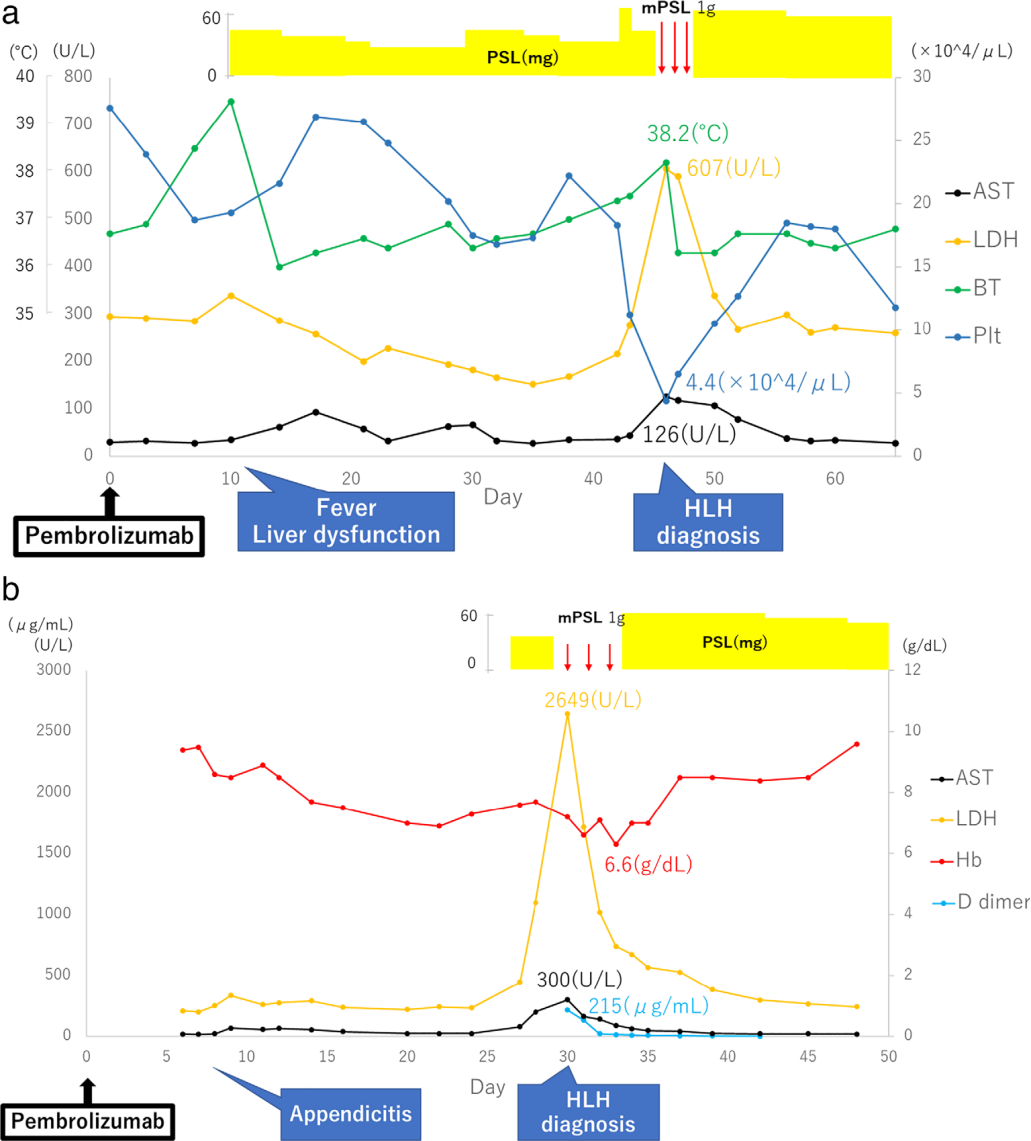
(a) Clinical course after administration of pembrolizumab in the first patient. AST, aspartate aminotransferase; BT, body temperature; HLH, hemophagocytic lymphohistiocytosis; LDH, lactate dehydrogenase; mPSL, methylprednisolone; Plt, platelet; PSL, prednisolone. (b) Clinical course after administration of the last pembrolizumab dose in the second patient. AST, aspartate aminotransferase; Hb, hemoglobin; HLH, hemophagocytic lymphohistiocytosis; LDH, lactate dehydrogenase; mPSL, methylprednisolone; PSL, prednisolone

He developed a fever, and his liver enzyme levels were elevated at 10 days after the first dose of pembrolizumab (200 mg). Corticotherapy was initiated to treat suspected immune‐related AEs, and his symptoms gradually improved. On day 46, after the first dose, he developed fever, cytopenia, coagulation abnormality, and elevated liver enzyme levels. We suspected HLH, and laboratory tests revealed a ferritin level of 11 273 ng/mL. Bone marrow aspiration showed hemophagocytic macrophages; his HScore was 175. No infections were detected. We initiated corticotherapy, and his blood test findings improved (Figure 1(a)).

### Case 2

A 60‐year‐old woman was diagnosed with stage IIIB lung adenocarcinoma. She was treated with concurrent chemoradiotherapy, and durvalumab was administered as consolidation therapy. We found brain metastasis in the cranial magnetic resonance imaging scan after 16 cycles of durvalumab. She underwent whole‐brain irradiation and chemotherapy. Six days after the second cycle of pemetrexed + pembrolizumab (200 mg), she developed acute appendicitis and underwent appendectomy. On day 30, after the last dose of pembrolizumab, she developed cytopenia, coagulation abnormalities, and elevated liver enzyme levels. We suspected HLH because of the elevated ferritin levels (64 726 ng/mL). Her HScore was 185, confirming HLH. After ensuring that the patient had no infections, we initiated corticotherapy, which significantly improved her laboratory findings (Figure 1(b)).

## DISCUSSION

The HLH‐2004 diagnosis criteria have been widely used for HLH, requiring five out of the eight criteria to be met for diagnosis (Table 1). However, there are several problems in diagnosing secondary HLH.^2^ First, the criteria were originally established for primary HLH and not validated for secondary HLH, which has been empirically diagnosed by experts. Second, the eight criteria are not weighted. Therefore, HLH may still be diagnosed by some experts despite five criteria not being met.^3^ Third, it is difficult to measure some criteria routinely, such as natural killer cell activity or soluble interleukin‐2 receptor levels. Recently, hyperferritinemia has been considered a strong indicator of HLH.^3^ Fardet et al. developed the HScore, which uses weighted criteria, to calculate HLH probability.^2^ The HScore cutoff value for HLH is 169 (sensitivity, 93%; specificity, 86%)^2^, ^3^ (Table 2). There has been no trial to determine whether the HLH‐2004 diagnosis criteria or HScore is superior. However, the high sensitivity and specificity of the HScore suggest its effectiveness at diagnosing HLH.

**TABLE 1:** HLH‐2004 diagnosis criteria

Fever ≥38.5°C
Splenomegaly
Cytopenia with at least two of the following (Hb <10 g/dL, Plt <100 000/μL, Neu <1000/μL)
TG >265 mg/dL and/or fibrinogen <150 mg/dL
Ferritin >500 ng/mL
sIL‐2R >age‐adjusted laboratory‐specific norms
Hemophagocytosis in bone marrow, spleen, lymph node, or liver
Low or absent NK cell activity

*Note*: Five of the eight criteria are needed to fulfill HLH diagnosis.

Abbreviations: Hb, hemoglobin; Neu, neutrophils; NK, natural killer; Plt, platelets; sIL‐2R, soluble interleukin‐2 receptor; TG, triglycerides.

**TABLE 2:** HScore

Known underlying immunosuppression	No (0), Yes (18)
Temperature (°C)	<38.4 (0), 38.4–39.4 (33), >39.4 (49)
Organomegaly	No (0), hepatomegaly or splenomegaly (23), hepatomegaly and splenomegaly (38)
Number of cytopenias	1 lineage (0), 2 lineages (24), 3 lineages (34)
Ferritin (ng/mL)	<2000 (0), 2000–6000 (35), >6000 (50)
Triglyceride (mg/dL)	<132.7 (0), 132.7–354 (44), >354 (64)
Fibrinogen (g/L)	>2.5 (0), ≤2.5 (30)
AST (U/L)	<30 (0), ≥30 (19)
Hemophagocytosis features on bone marrow aspirate	No (0), Yes (35)

*Note*: Cytopenia is defined as hemoglobin ≤9.2 mg/dL, white blood cell ≤5000/mm^3^, and platelets ≤110 000/mm^3^.

Abbreviation: AST, aspartate aminotransferase.

**FIGURE 2:**
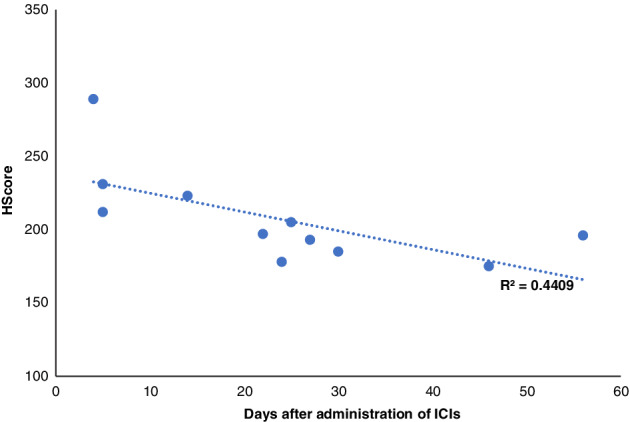
Relationship between HScore and days from administration of the last dose of immune checkpoint inhibitors. Late presentation of hemophagocytic lymphohistiocytosis (HLH) leads to a lower HScore (R^2^ = 0.4409)

Only 45 HLH cases have been reported to be caused by pembrolizumab, according to VigiBase, as of January 2021 (http://www.vigiaccess.org/). We reviewed 11 cases of ICI‐induced HLH, including our cases (Table 3).^4^, ^5^, ^6^, ^7^, ^8^, ^9^, ^10^, ^11^ The median interval from the last dose to HLH diagnosis was 24 days; therefore, the two patients discussed in our report were diagnosed in the late phase. Based on the time interval, cases in which HLH was diagnosed after a long interval showed a lower HScore than those diagnosed after a short interval (Figure 2). In almost all late‐HLH cases over 24 days, ICI treatment was interrupted to treat other immune‐related AEs using steroids, which may have alleviated the condition and HScore.

**TABLE 3:** Characteristics, HScore, and clinical courses of patients with ICI‐related hemophagocytic lymphohistiocytosis

Study	Age/sex	Day from last ICI	Steroid administration before HLH	HScore	Known underlying immunosuppression	BT	Organomegaly (liver/spleen)	Number of cytopenias	Ferritin	TG	Fibrinogen	AST	Bone marrow aspirate	Treatments	Clinical courses
Malissen et al. ^4^	42/M	5	No	212	Yes	–	–	–	–	–	–	–	–	Corticotherapy	Improved
Malissen et al. ^4^	81/M	5	No	231	Yes	–	–	–	–	–	–	–	–	Corticotherapy	Died
Michot et al. ^5^	52/F	56	Yes	196	Yes	N/A	N/A/N/A	3	>6000	>300	N/A	N/A	Yes	Dexamethasone and etoposide	Died
Kalmuk et al. ^6^	61/M	4	No	289	No	> 39.4	Yes/Yes	2	57 934	285	1.34	289	Yes	Dexamethasone and etoposide	Improved
Honjo et al. ^7^	52/F	14	No	223	Yes	38.6	N/A/N/A	2	3877	357	1.85	1556	N/A	Corticotherapy and MMF	Improved
Thummalapalli et al. ^8^	74/M	25	Yes	205	Yes	N/A	Yes/Yes	1	33 738	843	N/A	N/A	Yes	–	Died
Satzger et al. ^9^	26/F	22	Yes	197	Yes	>38.4	N/A/Yes	2	22 871	N/A	0.65	1100	N/A	Corticotherapy and MMF	Improved
Okawa et al. ^10^	78/M	24	Yes	178	Yes	>38.4	Yes/N/A	1	35 400	N/A	N/A	98	Yes	Corticotherapy	Improved
Akagi et al. ^11^	74/M	27	Yes	193	Yes	38.9	Yes/Yes	1	28 976	88	4.94	84	Yes	Dexamethasone and etoposide	Improved
Our first case	78/M	46	Yes	175	Yes	<38.4	Yes/N/A	1	11 273	N/A	0.643	126	Yes	Corticotherapy	Improved
Our second case	60/F	30	No	185	Yes	<38.4	No/No	2	64 726	139	2.07	300	N/A	Corticotherapy	Improved

*Note*: N/A is counted as a negative finding.

Abbreviations: AST, aspartate aminotransferase; BT, body temperature; ICI, immune checkpoint inhibitor; MMF, mycophenolate mofetil; N/A, not available; TG, triglyceride.

The mortality rate associated with HLH is 23%, which is one of the highest rates among immune‐related AEs^12^; the prognosis can be improved by early intervention in the case of Epstein–Barr virus (EBV)‐associated HLH.^13^ Late presentation cases of HLH are difficult to diagnose. However, our two cases were diagnosed promptly using the HScore over 24 days. We introduce a procedure for diagnosing secondary HLH caused by ICIs. HLH is suspected by the occurrence of cytopenia and marked inflammatory responses. If a patient had hyperferritinemia, we calculated the HScore to estimate HLH probability. This procedure will make it easier for any clinician to diagnose HLH, ensuring timely treatment.

Table 3 shows that cases with high HScore (>190) were improved not only by steroids but also by other immunosuppressive agents, suggesting that the treatment option may depend on HScore.

Only a few HLH cases have been reported. Additional cases are required to evaluate effectiveness of the HScore for patients with ICIs.

## CONFLICT OF INTEREST

The authors declare no conflict of interest.
